# *Lutzomyia longipalpis* Saliva Triggers Lipid Body Formation and Prostaglandin E_2_ Production in Murine Macrophages

**DOI:** 10.1371/journal.pntd.0000873

**Published:** 2010-11-02

**Authors:** Théo Araújo-Santos, Deboraci Brito Prates, Bruno Bezerril Andrade, Danielle Oliveira Nascimento, Jorge Clarêncio, Petter F. Entringer, Alan B. Carneiro, Mário A. C. Silva-Neto, José Carlos Miranda, Cláudia Ida Brodskyn, Aldina Barral, Patrícia T. Bozza, Valéria Matos Borges

**Affiliations:** 1 Centro de Pesquisas Gonçalo Moniz, FIOCRUZ-BA, Salvador, Brasil; 2 Universidade Federal da Bahia, Salvador, Brasil; 3 Laboratório de Imunofarmacologia, Instituto Oswaldo Cruz, Rio de Janeiro, Brasil; 4 Institutos de Bioquímica Médica, Universidade Federal do Rio de Janeiro, Rio de Janeiro, Brasil; 5 Instituto de Investigação em Imunologia, Instituto Nacional de Ciência e Tecnologia (INCT), São Paulo, Brasil; National Institute of Allergy and Infectious Diseases, United States of America

## Abstract

**Background:**

Sand fly saliva contains molecules that modify the host's hemostasis and immune responses. Nevertheless, the role played by this saliva in the induction of key elements of inflammatory responses, such as lipid bodies (LB, also known as lipid droplets) and eicosanoids, has been poorly investigated. LBs are cytoplasmic organelles involved in arachidonic acid metabolism that form eicosanoids in response to inflammatory stimuli. In this study, we assessed the role of salivary gland sonicate (SGS) from *Lutzomyia* (*L.*) *longipalpis*, a *Leishmania infantum chagasi* vector, in the induction of LBs and eicosanoid production by macrophages *in vitro* and *ex vivo*.

**Methodology/Principal Findings:**

Different doses of *L. longipalpis* SGS were injected into peritoneal cavities of C57BL/6 mice. SGS induced increased macrophage and neutrophil recruitment into the peritoneal cavity at different time points. Sand fly saliva enhanced PGE_2_ and LTB_4_ production by harvested peritoneal leukocytes after *ex vivo* stimulation with a calcium ionophore. At three and six hours post-injection, *L. longipalpis* SGS induced more intense LB staining in macrophages, but not in neutrophils, compared with mice injected with saline. Moreover, macrophages harvested by peritoneal lavage and stimulated with SGS *in vitro* presented a dose- and time-dependent increase in LB numbers, which was correlated with increased PGE_2_ production. Furthermore, COX-2 and PGE-synthase co-localized within the LBs induced by *L*. *longipalpis* saliva. PGE_2_ production by macrophages induced by SGS was abrogated by treatment with NS-398, a COX-2 inhibitor. Strikingly, SGS triggered ERK-1/2 and PKC-α phosphorylation, and blockage of the ERK-1/2 and PKC-α pathways inhibited the SGS effect on PGE_2_ production by macrophages.

**Conclusion:**

In sum, our results show that *L. longipalpis* saliva induces lipid body formation and PGE_2_ production by macrophages *ex vivo* and *in vitro* via the ERK-1/2 and PKC-α signaling pathways. This study provides new insights regarding the pharmacological mechanisms whereby *L. longipalpis* saliva influences the early steps of the host's inflammatory response.

## Author Summary

After the injection of saliva into the host's skin by sand flies, a transient erythematous reaction is observed, which is related to an influx of inflammatory cells and the release of various molecules that actively facilitate the blood meal. It is important to understand the specific mechanisms by which sand fly saliva manipulates the host's inflammatory responses. Herein, we report that saliva from *Lutzomyia (L.) longipalpis*, a widespread *Leishmania* vector, induces early production of eicosanoids. Intense formation of intracellular organelles called lipid bodies (LBs) was noted within those cells that migrated to the site of saliva injection. *In vitro* and *ex vivo*, sand fly saliva was able to induce LB formation and PGE_2_ release by macrophages. Interestingly, PGE_2_ production induced by *L. longipalpis* saliva was dependent on intracellular mechanisms involving phosphorylation of signaling proteins such as PKC-α and ERK-1/2 and subsequent activation of cyclooxygenase-2. Thus, this study provides new insights into the pharmacological properties of sand fly saliva and opens new opportunities for intervening with the induction of the host's inflammatory pathways by *L. longipalpis* bites.

## Introduction

To obtain a blood meal, sand flies locate blood by introducing their mouthparts into the vertebrate host's skin, tearing tissues, lacerating capillaries and creating hemorrhagic pools upon which they feed. During this process, sand flies need to circumvent a number of the host's homeostatic responses, such as activation of blood coagulation cascades, vasoconstriction, platelet aggregation and immune responses [1], [2]. In this environment, sand flies evolved an array of potent pharmacologic components with redundant and synergistic activities that subvert the host's physiological responses and favor the blood meal. Intense research using high-throughput analyses has been conducted to identify salivary factors and their biological activities. *Lutzomyia* (*L.*) *longipalpis*, the main vector of visceral leishmaniasis in South America, has been extensively studied. During the inflammatory response, *L. longipalpis* saliva induces cellular recruitment, modulates both antibody production and the formation of immunocomplexes [3], [4], regulates T cell activities and inhibits dendritic cells and macrophages, the latter being preferential host cells for *Leishmania* [5], [6]. There is also evidence that maxadilan, a *L. longipalpis* salivary protein with vasodilator properties, down-regulates LPS-induced TNF-α and NO release through a mechanism dependent on PGE_2_ and IL-10 [7].

PGE_2_ is an eicosanoid derived from arachidonic acid (AA) metabolism by the enzyme cyclooxygenase (COX). Prostanoids and leukotrienes can be intensely produced by macrophages during inflammatory responses [8], and these mediators are implicated in cellular recruitment and activation. Among the eicosanoids, LTB_4_ induces neutrophil recruitment [9], whereas PGE_2_ and PGD_2_ attract mainly macrophages [10]. Previous studies used different experimental models to show that *L. longipalpis* saliva induces an influx of neutrophils [11] and macrophages [12], but neither the role of saliva in LTB_4_ and PGE_2_ release nor the involvement of these mediators in this process has been fully addressed.

Under inflammatory and infectious conditions, prostaglandins and others lipid mediators are mainly produced by cytoplasmic organelles called lipid bodies (LB) [13]. Intense research over the past few years has defined lipid bodies as dynamic cytoplasmic organelles. It has been demonstrated that lipid bodies compartmentalize enzymes involved in the biosynthesis, transport and catabolism of lipids, proteins involved in membrane and vesicular transport and proteins involved in cell signaling and inflammatory mediator production, including eicosanoid-forming enzymes, phospholipases and protein kinases. All of these molecules can be localized into lipid bodies in various cells under a range of activation conditions, suggesting a wide role for lipid bodies in the regulation of cellular lipid metabolism and signaling [13].

Herein, we evaluated the effect of *L. longipalpis* salivary gland sonicate (SGS) on the induction of LB formation as well as PGE_2_ and LTB_4_ production *in vitro* and *ex vivo*. Moreover, we explored the role of peritoneal macrophages in the production of these lipid mediators in response to *L. longipalpis* SGS *in vitro*. Finally, we found that the PGE_2_ production induced by *L. longipalpis* saliva is dependent on intracellular mechanisms involving the phosphorylation of signaling proteins such as PKC-α and ERK-1/2 and subsequent activation of COX-2.

## Methods

### Antibodies and Reagents

Dimethylsulfoxide (DMSO) was purchased from ACROS Organics (New Jersey, NJ). RPMI 1640 medium and L-glutamine, penicillin, and streptomycin were from Invitrogen (Carlsbad, CA). Nutridoma-SP was from Roche (Indianapolis, IN). A23187 calcium ionophore, was from Calbiochem/Novabiochem Corp. (La Jolla, CA). NS-398, PGE_2_ and LTB_4_ enzyme-linked immunoassay (EIA) Kits, anti-murine COX-2 and PGE-synthase antibodies were all from Cayman Chemical (Ann Arbor, MI). 4,4-difluoro-1,3,5,7,8-pentamethyl-4-bora-3a,4a-diaza-s-indacene (BODIPY 493/503) was obtained from Molecular Probes (Eugene, OR). Osmium tetroxide (OsO4) was obtained from Electron Microscopy Science (Fort Washington, PA). Aqua Polymount was from Polysciences (Warrington, PA). Thiocarbohydrazide, Ca^2+^-Mg^2+^-free HBSS(^−/−^), HBSS(^+/+^) with Ca^2+^-Mg^2+^, LPS from *Escherichia coli* (serotype 0127:b8), and *N*-ethyl-*N*'-(3-dimethylaminopropyl) carbodiimide hydrochloride (EDAC) were purchased from Sigma-Aldrich (St. Louis, MO). Rabbit anti-mouse kinase proteins were from Santa Cruz Biotechnology (Santa Cruz, CA). PD 98059, 2′-Amino-3′-methoxyflavone and Bisindolylmaleimide-I, 2-[1-(3-Dimethylaminopropyl)-1H-indol-3-yl]-3-(1H-indol-3-yl)-maleimide were obtained from Merck-Calbiochem (Darmstadt, Hessen).

### Mice

Inbred male C57BL/6 mice, age 6–8 weeks, were obtained from the animal facility of Centro de Pesquisas Gonçalo Moniz, Fundação Oswaldo Cruz (CPqGM-FIOCRUZ, Bahia, Brazil). All experimental procedures were approved and conducted according to the Animal Care and Using Committee of the FIOCRUZ.

### Sand flies and preparation of salivary glands

Adult *Lutzomyia longipalpis* captured in Cavunge (Bahia, Brazil) were reared at the Laboratório de Imunoparasitologia/CPqGM/FIOCRUZ (Bahia, Brazil) as described previously [3]. Salivary glands were dissected from 5- to 7-day-old *L. longipalpis* females under a Stemi 2000 Carl Zeiss stereoscopic microscope (Göttingen, Germany) and stored in groups of ten pairs in 10 µL of endotoxin-free PBS at −70°C. Immediately before use, the glands were sonicated with a Branson Sonifier 450 (Danbury, CT) and centrifuged at 10,000× g for four minutes. The supernatant from salivary gland sonicate (SGS) was used for experiments. The level of LPS contamination of *L. longipalpis* SGS preparations was determined using a commercially available LAL Chromogenic Kit (Lonza Bioscience, Walkersville, MD); negligible levels of endotoxin were found in the salivary gland supernatant (0.1 ηg/mL). We measured 0.7 micrograms of protein in an amount equivalent to 0.5 pair of salivary glands and used SGS dilutions (2.0–0.2 pairs) in our experiments [14].

### Leukocyte recruitment to the peritoneal cavity

To assess the leukocyte recruitment induced by *L. longipalpis* SGS, we used the well-established peritoneal model of inflammation because the peritoneal cavity is a self-contained and delineated compartment and thus provides a large number of post-stimulus leukocytes. As previously established in the air pouch murine model [12] and peritoneal cavity (unpublished data), a 0.5-pair dose of SGS was used for the leukocyte recruitment assay. C57BL/6 mice were inoculated i.p. with 0.1 mL of *L. longipalpis* SGS (0.5 pair/cavity), endotoxin-free saline (negative control) or 0.1 mL of LPS (20 µg/mL, positive control). At 1, 3 and 6 h post-stimulus, leukocytes inside the peritoneal cavity were harvested by injection and recovery of 10 mL of endotoxin-free saline. Total counts were performed on a Neubauer hemocytometer after staining with Turk's solution. Differential cell counts (200 cells total) were carried out microscopically on cytospin preparations stained with Diff-Quick.

### Lipid body staining and quantification

Cells harvested by peritoneal lavage 1, 3, 6 or 24 h after i.p. injection of 0.1 mL of *L. longipalpis* SGS (0.5 pair/cavity), endotoxin-free saline or LPS (20 µg/mL) were centrifuged at 400× *g* and the lipid bodies within the leukocytes were stained with BODIPY 493/503 (5 ug/mL) according to Plotkowisk *et al.* [15]. Samples were analyzed using a FACSort flow cytometer from Becton Dickinson Immunocytometry Systems (San Jose, CA) and by fluorescence microscopy.

Macrophages adhered to coverslips within 24-well plates were fixed with 3.7% formaldehyde and stained with osmium tetroxide as described previously [16]. The morphology of the fixed cells was observed, and lipid bodies were counted by light microscopy with a 100x objective lens in 50 consecutively scanned macrophages.

### Resident peritoneal macrophage harvesting and treatments

For *in vitro* assays, macrophages were obtained by peritoneal lavage with cold RPMI 1640. Then, cells were centrifuged at 400× g for 10 minutes. Macrophages (3×10^5^/well) were cultured in 1 mL of RPMI 1640 medium supplemented with 1% Nutridoma-SP, 2 mM L-glutamine, 100 U/mL penicillin and 100 µg/mL streptomycin in 24-well plates for 24 hours. Next, the macrophages were stimulated with different doses of *L. longipalpis* SGS (0.2, 0.5, 1.0, 1.5, 2.0 pairs/well). In some experiments, LPS (500 ng/well) was used as a positive control. One, 6, 24, 48 and 72 hours after stimuli, supernatants were collected and cells were fixed with 3.7% formaldehyde. For inhibitory assays, macrophages were pretreated for one hour with 1 μM NS-398, a COX-2 inhibitor; 20 ηM BIS, a PKC inhibitor; or 50 μM PD98059, an ERK-1/2 inhibitor. Then, the cells were stimulated with SGS (1.5 pairs/well) or medium containing vehicle (DMSO) for 24 hours, and the supernatants were collected for eicosanoid measurement. Cell viability as assessed by trypan blue exclusion was always greater than 95% after the end of treatment.

### Immunofluorescence for COX-2 and PGE-synthase

Resident peritoneal macrophages were cultured on coverslips in the presence of *L. longipalpis* SGS (1.5 pair/well) as described above. After 24 h, the cells were washed twice with 500 µl of HBSS^−/−^ and immediately fixed with 500 µL of water-soluble EDAC (1% in HBSS^−/−^), used to cross-link eicosanoid carboxyl groups to amines in adjacent proteins. After 15 min of incubation at room temperature (RT) with EDAC to promote both cell fixation and permeabilization, macrophages were then washed with HBSS^−/−^ and incubated with 1 µM BODIPY 493/503 for 30 min. Then, the cover slips were washed with HBSS^−/−^ and incubated with mouse anti-COX-2 (1∶150) or anti-PGE-synthase (1∶150) for 1 h at RT. MOPC 21 (IgG1) was used as a control. After further washes, cells were incubated with biotinylated goat anti-rabbit IgG secondary Ab, washed twice and incubated with avidin conjugated with PE for 30 min. The cover slips were then washed three times and mounted in Vectashield medium containing DAPI (Vector Laboratories, Burlingame, CA). The samples were observed by fluorescence microscopy and images were acquired using the software Image-Pro Plus (Media Cybernetics, Silver Spring, MD).

### Western blotting analysis

Macrophages were treated or not with SGS (1.0 pair/well) for 40 min. Next, the cells were washed once with phosphate-buffered saline, homogenized in lysis buffer containing phosphatase inhibitors (10 mM TRIS-HCl, pH 8.0, 150 mM NaCl, 0.5% v/v Nonindet-P40, 10% v/v glycerol, 1 mM DTT, 0.1 mM EDTA, 1 mM sodium orthovanadate, 25 mM NaF and 1 mM PMSF) and a protease inhibitor cocktail (Roche, Indianapolis, IN). Protein concentrations were determined using the method of Lowry *et al.* [17] with BSA as the standard. Total proteins (20 µg) were then separated by 10% sodium dodecyl sulfate–polyacrylamide gel electrophoresis (SDS–PAGE) as described previously [18] and transferred onto nitrocellulose membranes. The membranes were blocked in Tris-buffered saline (TBS) supplemented with 0.1% Tween 20 (TT) plus 5% BSA for 1 h before incubation overnight in the primary rabbit anti-mouse PKC-α and anti-ERK-1/2 (1∶1,000) antibodies. After removal of the primary antibody and washing five times in TT, the membranes were incubated in the secondary antibody conjugated to peroxidase (1∶10,000) for 1 h. Washed blots were then incubated with an ECL chemiluminescence kit (Amersham, UK). The membranes were discharged and immunoblotted again using primary rabbit anti-mouse phosphorylated-PKC-α and ERK-1/2 (1∶1,000) antibodies according to the manufacturer's instructions (Amersham, UK).

Quantification of the level of proteins in the western blotting membranes was determined by densitometry. Briefly, bands were scanned and processed using Adobe Photoshop 5.0 software (Adobe Systems Inc.), and arbitrary values for protein density were estimated. Ratios between phosphorylated and unphosphorylated proteins were obtained to calculate the difference between groups.

### PGE_2_ and LTB_4_ measurement

C57BL/6 mice were inoculated i.p. with 0.1 mL of *L. longipalpis* SGS (0.5 pair/cavity), endotoxin-free saline or 0.1 mL of LPS (500 ηg/mL). At 1, 3 and 6 h post-stimulus, leukocytes were harvested by peritoneal washing with HBSS^−/−^ and 1×10^6^ cells/mL were resuspended in HBSS^+/+^ and stimulated with A23187 (0.5 µM) for 15 min [16]. The reactions were stopped on ice, and the samples were centrifuged at 500× g for 10 min at 4°C. Supernatants from leukocytes re-stimulated *ex vivo* or those of *in vitro* assays were collected for measurement of PGE_2_ and LTB_4_ by enzyme-linked immunoassay (EIA) according to the manufacturer's instructions (Cayman Chemical, Ann Arbor, MI).

### Statistical analysis

The *in vivo* assays were performed using at least five mice per group. Each experiment was repeated at least three times. Data are reported as the mean and standard error of representative experiments and were analyzed using GraphPad Prism 5.0 software. Disparities in leukocyte recruitment, lipid bodies and lipid mediator quantification were explored using Student's *t* test. Means from different groups from the *in vitro* assays were compared by ANOVA followed by Bonferroni's test or a post-test for linear trends. Differences were considered statistically significant when *p*≤0.05.

## Results

### Lipid bodies and eicosanoids in leukocytes recruited by *L. longipalpis* SGS

**Figure 1:**
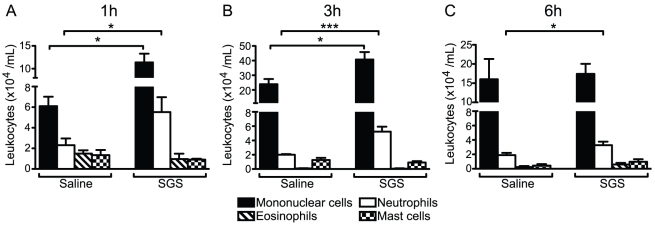
Leukocyte influx into the peritoneal cavity of C57BL/6 mice in response to *L. longipalpis* SGS. Mice were injected i.p. with endotoxin-free saline or SGS (0.5 pair/cavity). One (A), 3 (B) and 6 (C) hours after stimulation, cells were harvested by peritoneal lavage and differential leukocyte counts were performed on Diff-quick stained cytospin preparations. The data are the means and SEM from an experiment representative of three independent experiments. Groups were compared using Student's *t* test at each time point. *, *p*<0.05 and ***, *p*<0.001.

**Figure 2:**
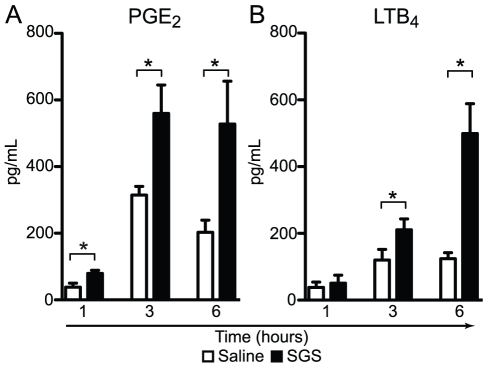
Kinetics of eicosanoid production in response to *L. longipalpis* SGS *ex vivo*. C57BL/6 mice were injected i.p. with saline or SGS (0.5 pair/cavity). One, 3 and 6 hours after stimulation, peritoneal cavities were washed and cells were harvested. The cells were then incubated with A23187 (0.5 µM) for 15 min at 37°C to evaluate LTB_4_ and PGE_2_ production. The concentrations of PGE_2_ (A) and LTB_4_ (B) in the supernatant were measured by ELISA. The data are the means and SEM from an experiment representative of three independent experiments. Groups were compared using Student's *t* test at each time point. *, *p*<0.05.

To measure the leukocyte recruitment induced by SGS, we injected 100 μL of saline or SGS (0.5 pair/cavity), and 1, 3 and 6 hours after injection, we enumerated total leukocytes recruited to the peritoneal cavity. Most of the cells recruited were mononuclear cells and neutrophils (Figure 1). In this context, SGS induced mononuclear cell recruitment for 3 hours (Figure 1 A and B) and neutrophil recruitment for over 6 hours (Figure 1A–C) of stimulation when compared with the saline group. Other cell populations (eosinophils and mast cells) were not altered after SGS stimulation, and there was no variation in these numbers over time (Figure 1). The peritoneal cell population in unstimulated animals (time zero) was composed of mononuclear cells (2.985×10^4^ ±0.027) and negligible amounts of neutrophils (0.018×10^4^ ±0.027). At this time, macrophages are the major cells within the mononuclear population in the peritoneal cavity besides lymphocytes, which represent ∼10% of mononuclear cells (data not shown). As shown in Figure 2, SGS administration led to enhanced PGE_2_ (Figure 2A) and LTB_4_ (Figure 2B) release within those cells recruited to the peritoneal cavity.

**Figure 3:**
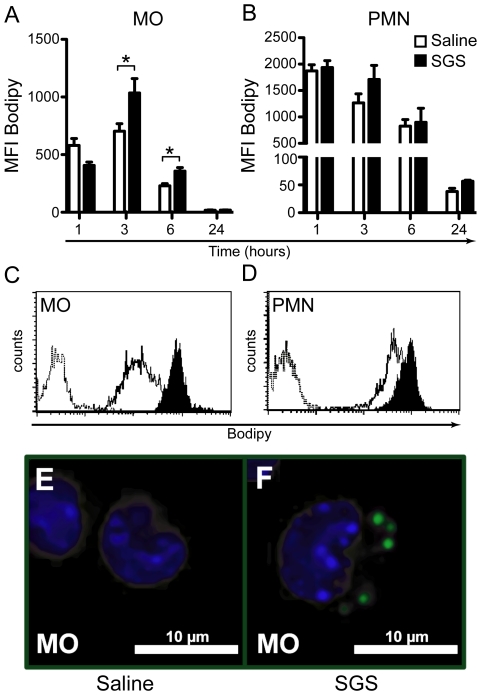
Lipid body formation induced by SGS *in vivo*. C57BL/6 mice were injected i.p. with saline or SGS (0.5 pair/cavity). One, 3, 6 and 24 hours after stimulation, cells were harvested from the peritoneal cavity and stained with the neutral lipid probe BODIPY 493/503. Kinetics of LB formation in mononuclear (A) and polymorphonuclear (B) cells. Mean fluorescence intensity (MFI) histograms of mononuclear (C) and polymorphonuclear (D) cell populations at the 3-hour time point. Dotted lines indicate unstained cells, full lines indicate stained cells from the saline group (empty curves) and from the SGS-treated group (filled curves). LBs in mononuclear cells stimulated with saline (E) or SGS (F) for 3 h detected by fluorescence microscopy, nuclei stained with DAPI. Groups were compared using Student's *t* test at each time point. *, *p*<0.05. MO, mononuclear; PMN, polymorphonuclear.

Because LBs are sites of eicosanoid production [19], we evaluated LB formation in leukocytes recruited to the peritoneal cavity by FACs using the neutral lipid probe BODIPY 493/503. The kinetics of LB formation was evaluated at 1, 3, 6 and 24 hours after SGS stimulation by measuring mean fluorescence intensity (MFI). SGS increased MFI in mononuclear but not in polymorphonuclear cells after 3 and 6 hours, (Figure 3A and B) compared with the saline group. Histograms (Figure 3C and D) and fluorescence microscopic images (Figures 3E and F) at the 3-hour time point confirmed these effects of SGS on macrophages.

### *L. longipalpis* SGS triggers LB biogenesis in peritoneal macrophages *in vitro*

**Figure 4:**
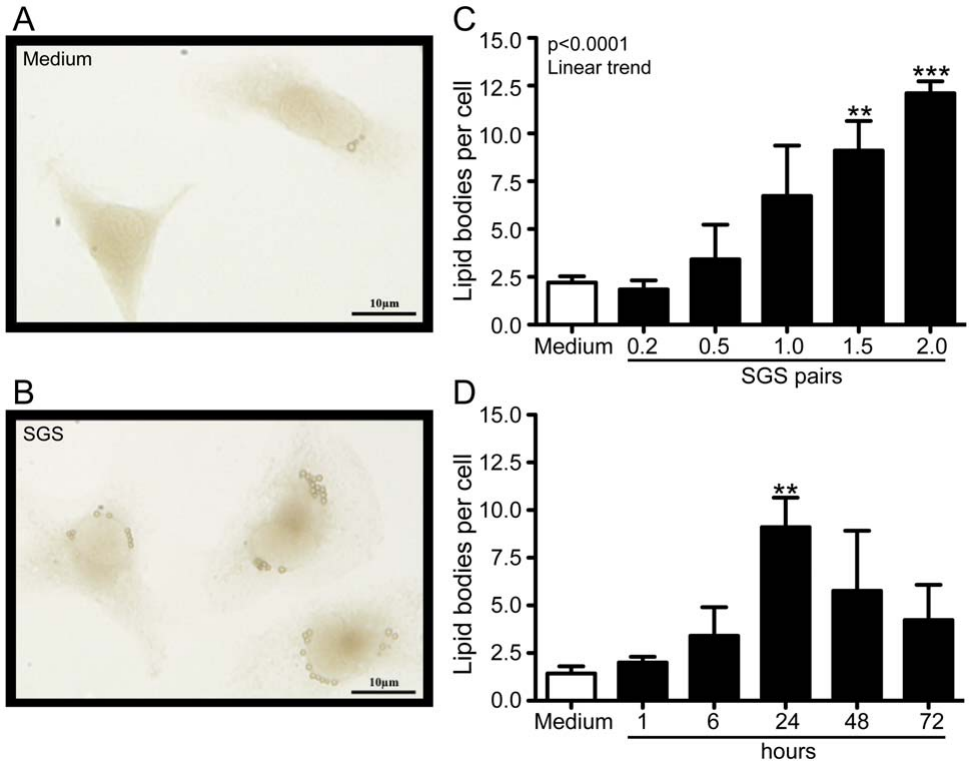
Effect of *L. longipalpis* SGS on lipid body formation in peritoneal macrophages *in vitro*. Representative image of peritoneal macrophages untreated (A) or stimulated with SGS (1.5 pair/well) (B) for 24 hours. Dose-response (C) and kinetics (D) of lipid body formation induced by SGS in peritoneal macrophages. **, *p*<0.01 and ***, *p*<0.001 compared with unstimulated cells.

To assess the role of SGS in lipid body formation in resident macrophages, we stimulated these cells with different doses of SGS (0.2–2.0 pairs/well) for different time periods (1, 6, 24, 48 and 72 hours). At 24 hours post-stimulus, SGS strongly induced LB formation compared with the untreated group (Figure 4A–D). LB formation was induced in a dose-dependent manner, and the maximum of LBs per macrophage was observed at a dose of 2.0 pairs/well (Figure 4C). Because LB formation induced by SGS (1.5 pairs/well) was more evident at 24 hours (Figure 4D), we selected this time point to perform further experiments.

### *L. longipalpis* SGS induces macrophage PGE_2_ production via the COX-2 enzyme

**Figure 5:**
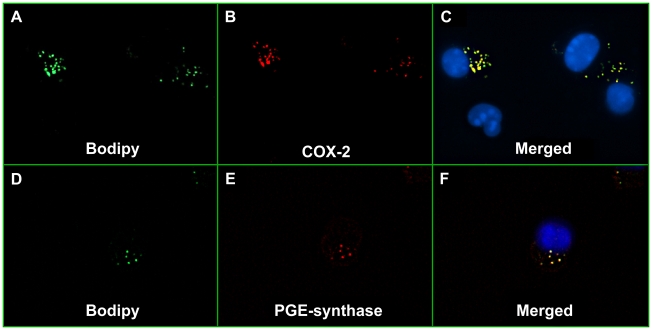
COX-2 and PGE-synthase co-localize within lipid bodies induced by *L. longipalpis* SGS. Peritoneal macrophages were stimulated with SGS (1.5 pair/well) for 24 hours. BODIPY probe-labeled lipid bodies were visualized as green punctuate intra-cytoplasmic inclusions (A and D). COX-2 (B) and PGE-synthase (E) were localized with anti-COX-2 and anti- PGE-synthase antibodies, respectively. Merged images show co-localization of COX-2 (C) and PGE-synthase (F) within lipid bodies.

Prostaglandins are produced by cyclooxygenases, which occur in constitutive (COX-1) and inducible (COX-2) forms [20]. We investigated the expression and subcellular localization of COX-2 within SGS-stimulated macrophages. Immunofluorescence microscopy revealed the presence of COX-2 (Figure 5A–C) and PGE-synthase (Figure 5D–F) within LBs in macrophages stimulated with SGS.

**Figure 6:**
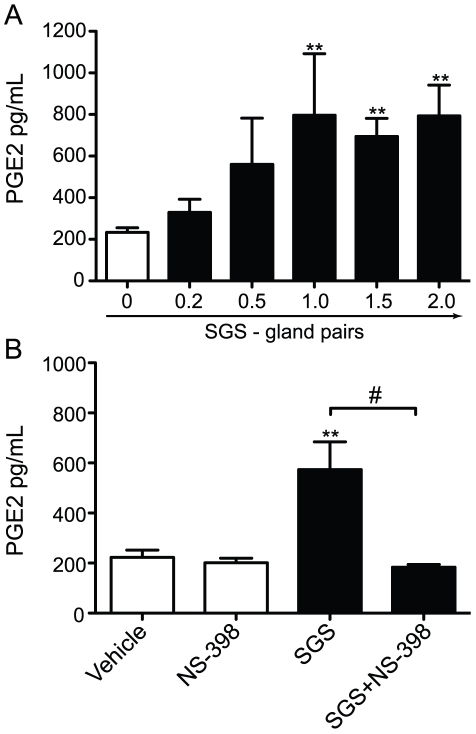
*L. longipalpis* SGS induces PGE_2_ production via COX-2. A, Dose-response of PGE_2_ production induced by SGS in peritoneal macrophages. B, Macrophages were pre-treated for 1 hour with the COX-2 inhibitor N-398 before incubation with SGS (1.5 pair/well). Twenty-four hours after stimulation, PGE_2_ was measured in the supernatant. The data are the means and SEM from a representative experiment of three independent experiments. **, *p*<0.01 and ^#^, *p*<0.05.

Next, we measured PGE_2_ and LTB_4_ production in the supernatant of macrophage cultures. SGS induced PGE_2_ production starting at 1.0 pair/well (Figure 6A), whereas LTB_4_ was not detectable under any conditions (data not shown). As expected, PGE_2_ production by macrophages stimulated with SGS was reduced to basal levels when the cells were pre-incubated with NS-398, a COX-2 inhibitor (Figure 6B). Thus, the PGE_2_ production in peritoneal macrophages induced by SGS occurs in newly formed lipid bodies and is dependent on COX-2.

### SGS induces PGE_2_ production via PKC-α and ERK-1/2

**Figure 7:**
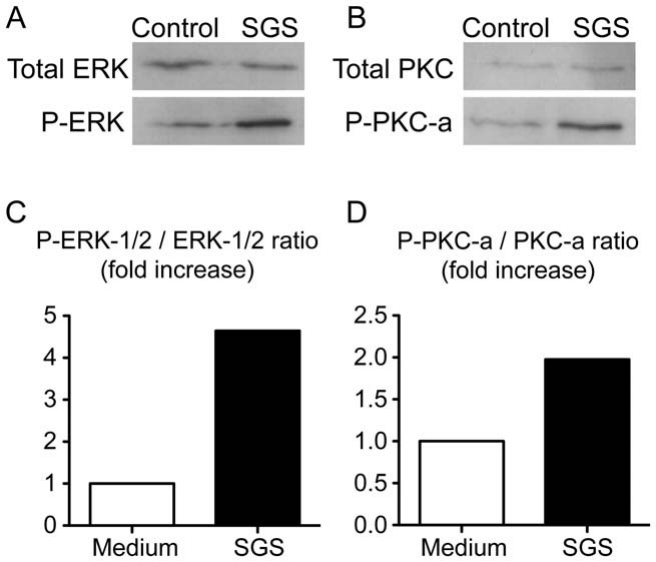
*L. longipalpis* SGS induces PKC-α and ERK phosphorylation. Peritoneal macrophages were incubated in the absence (control) or presence of SGS (1.5 pair/mL) for 40 min. The cells were lysed and immunoblotted using polyclonal anti-ERK-1/2 (A) or anti-PKC-α (B) antibodies. The membranes was discharged and immunoblotted using polyclonal anti- phospho-ERK-1/2 (A) or anti- phosphor-PKC-α (B) antibodies. Quantification of phosphorylated-ERK-1/2 (C) and phosphorylated-PKCα (D) was determined by densitometry. The data show the fold increase in the phosphorylated/unphosphorylated kinase ratio of the SGS group relative to the control group. P-, phosphorylated.

**Figure 8:**
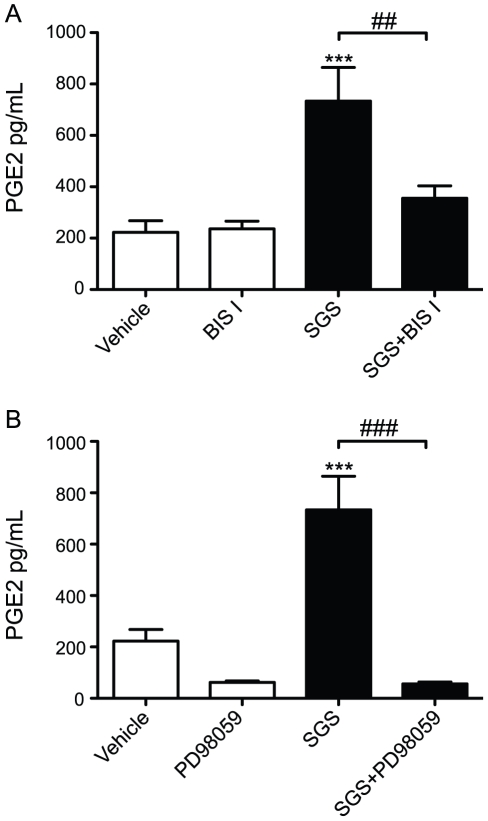
ERK and PKC kinase inhibitors abrogate PGE_2_ production induced by *L. longipalpis* SGS. Peritoneal macrophages were pre-treated for 1 hour with BIS I (A) or PD98059 (B) before incubation with SGS (1.5 pair/well). Twenty-four hours after stimulation, PGE_2_ was measured in the supernatant. The data are the mean and SEM from an experiment representative of three independent experiments. ***, *p*<0.001; ^##^, *p*<0.01 and ^###^, *p*<0.001. PD98059, ERK inhibitor; BIS-I, PKC inhibitor.

Multiple pathways are involved in the signaling for PGE_2_ production [13]. Recently, ERK and PKC-α were shown to be involved in COX-2 activity [21]. We observed that SGS activated both ERK (Figure 7A and C) and PKC-α phosphorylation (Figure 7B and D), but it did not alter the levels of the unphosphorylated proteins. To investigate whether these kinases are involved in the induction of PGE_2_ production by SGS, we pretreated macrophages with bisindolylmaleimide I (BIS I) and PD98059, PKC-α and ERK-1/2 inhibitors, respectively (Figure 8A–B). Inhibition of both enzymes completely abrogated PGE_2_ production induced by SGS (Figure 8A–B). In sum, these results suggest that PKC-α and ERK-1/2 are involved in the PGE_2_ production induced by SGS.

## Discussion

Sand fly saliva triggers an inflammatory response characterized by cellular influx followed by hemostatic and immune mechanism suppression. Nevertheless, the role of sand fly saliva in eicosanoid production during the early steps of the innate immune response is poorly understood. In inflammatory conditions, eicosanoids are mostly produced in cytoplasmic organelles called lipid bodies (LBs), which are formed in leukocytes and other cells involved in the inflammatory and infectious responses to several stimuli [13]. Herein, we showed that *L. longipalpis* saliva induces lipid body formation and PGE_2_ production in peritoneal macrophages *ex vivo* and *in vitro* via kinase phosphorylation and COX-2 activation.

Previous investigations have demonstrated that sand fly saliva plays an important role in cellular recruitment in multiple experimental models [3], [9], [11], [12], including *in vivo* sand fly bites [22]. Herein, we confirmed previous reports that *L. longipalpis* SGS induces an inflammatory infiltration composed mainly of macrophages and neutrophils. Moreover, we showed that the cellular recruitment induced by *L. longipalpis* saliva is concomitant with PGE_2_ and LTB_4_ production. In this scenario, lipid mediators could be triggering cellular recruitment. Secretion of LTB_4_ by resident macrophages plays an important role in neutrophil migration [23]. In addition, lipopolysaccharides induce macrophage migration via prostaglandin D_2_ and prostaglandin E_2_ [10].

Prostaglandin E_2_ is an abundant eicosanoid produced by inflammatory cells, and it is known to exert anti-inflammatory and vasodilator effects. PGE_2_ is found in *Ixodes scapularis* saliva and is also implicated in the immunomodulatory activity of tick saliva on dendritic cell and macrophage activation [24]. Furthermore, previous studies using saliva from several *Phlebotomus* species have suggested that the anti-inflammatory properties of sand fly saliva could be attributed to PGE_2_ and IL-10 released by dendritic cells [9], [25]. In these studies, the cellular recruitment induced by OVA stimulation was abrogated by saliva from various sand fly species [9], [25], which was associated with an anti-inflammatory profile dependent on the production of IL-10, IL-4 [25] and PGE_2_ [9]. Intriguingly, maxadilan, a vasodilator peptide with immunomodulatory activities present in *L. longipalpis* saliva, is able to induce LPS-activated macrophages to release PGE_2_ via COX-1, an enzyme that is constitutively active [7]. In the present study, we showed that *L. longipalpis* SGS triggers PGE_2_ production in resident macrophages by an inducible pathway, since this effect was completely abrogated when the cells were incubated in the presence of NS-398, a COX-2 inhibitor. Nevertheless, whether sand fly saliva contains other molecules involved in PGE_2_ production or pharmacological amounts of this mediator similarly to tick saliva remains unknown.

Our study is the first to establish a direct link between *L. longipalpis* saliva, eicosanoid production and lipid body formation. Under inflammatory and infectious conditions, lipid mediators are mainly produced within LBs, which compartmentalize both the substrate and the enzymatic machinery required for eicosanoid production [13]. In this regard, the enzymes COX and 5-LO have been localized to lipid bodies in various inflammatory cells by the use of multiple techniques including fluorescence microscopy [13]. Previous studies have shown that various inflammatory and infectious stimuli are able to trigger LB formation in macrophages [13], [19]. Our findings demonstrate that SGS induces LB formation in macrophages *in vivo* and *in vitro*, suggesting that *L. longipalpis* saliva acts directly on these cells, but not on neutrophils. Indeed, *L. longipalpis* SGS triggered LB formation in macrophages committed to PGE_2_ production via COX-2 and PGE-synthase.

Data regarding the direct effects of sand fly salivary compounds on host signaling pathways cells are scarce. The extracellular signal-regulated kinases (ERKs) and protein kinase C (PKC) are among the key enzymes implicated in signaling pathways of diverse cellular responses, including eicosanoid production. The MAP kinases ERK1 and ERK2 induce activation of cPLA2, an enzyme that hydrolyzes arachidonic acid, which is metabolized to prostaglandin H2 by COX [13]. Previous studies have demonstrated the compartmentalization of MAP kinases and cPLA2 at arachidonate-enriched lipid bodies [26], [27], as well as COX-2 and PGE-synthase [16], [28], [29]. Herein, it is shown for the first time that *L. longipalpis* SGS triggers ERK-1/2 and PKC-α phosphorylation in macrophages. Other studies have shown that COX-2 activation and PGE_2_ production in LPS stimulated-macrophages is dependent on the phosphorylation of protein kinases such as PKC-α [21] and ERK-1/2 [30]. We showed that the PGE_2_ production induced by SGS is dependent on both ERK-1/2 and PKC. This association between the activation of kinases and the metabolism of eicosanoids within lipid bodies may serve to enhance rapid eicosanoid production in response to extracellular stimuli such as sand fly saliva. Of note, in addition to their role in regulating the host response to infection by modulating inflammatory mediator production, lipid bodies may also serve as rich sources of nutrients for intracellular pathogens, thus favoring intracellular pathogen replication [31], [32].

In brief, the present work provides new insights into the mechanisms involved in macrophage responses to *L. longipalpis* saliva, including LB formation and the signaling pathways that trigger PGE_2_ release. Although the roles of the newly formed LBs and PGE_2_ induced by sand fly saliva in the pathogenesis of leishmaniasis have not yet been addressed, several studies have shown that PGE_2_ is essential to the infection of macrophages [33], [34] and parasite dissemination after infection [35]. The induction of PGE_2_ production by sand fly saliva demonstrated herein can influence the initial steps of host infection by favoring less intense macrophage activation. Our group and others have been providing strong evidence that saliva components are immunogenic and have potential as markers of exposure to sand fly vectors [36]–[39]. Further studies are required to determinate if the immunization based on components of vector saliva interferes in eicosanoid production with consequences for the host's immune response and the transmissibility of the parasite.

## References

[1] Andrade BB, Teixeira CR, Barral A, Barral-Netto M (2005). Haematophagous arthropod saliva and host defense system: a tale of tear and blood.. An Acad Bras Cienc.

[2] Peters NC, Sacks DL (2009). The impact of vector-mediated neutrophil recruitment on cutaneous leishmaniasis.. Cell Microbiol.

[3] Silva F, Gomes R, Prates D, Miranda JC, Andrade B (2005). Inflammatory cell infiltration and high antibody production in BALB/c mice caused by natural exposure to Lutzomyia longipalpis bites.. Am J Trop Med Hyg.

[4] Vinhas V, Andrade BB, Paes F, Bomura A, Clarencio J (2007). Human anti-saliva immune response following experimental exposure to the visceral leishmaniasis vector, Lutzomyia longipalpis.. Eur J Immunol.

[5] Costa DJ, Favali C, Clarencio J, Afonso L, Conceicao V (2004). Lutzomyia longipalpis salivary gland homogenate impairs cytokine production and costimulatory molecule expression on human monocytes and dendritic cells.. Infect Immun.

[6] Zer R, Yaroslavski I, Rosen L, Warburg A (2001). Effect of sand fly saliva on Leishmania uptake by murine macrophages.. Int J Parasitol.

[7] Soares MB, Titus RG, Shoemaker CB, David JR, Bozza M (1998). The vasoactive peptide maxadilan from sand fly saliva inhibits TNF-alpha and induces IL-6 by mouse macrophages through interaction with the pituitary adenylate cyclase-activating polypeptide (PACAP) receptor.. J Immunol.

[8] Wilborn J, DeWitt DL, Peters-Golden M (1995). Expression and role of cyclooxygenase isoforms in alveolar and peritoneal macrophages.. Am J Physiol.

[9] Carregaro V, Valenzuela JG, Cunha TM, Verri WA, Grespan R (2008). Phlebotomine salivas inhibit immune inflammation-induced neutrophil migration via an autocrine DC-derived PGE2/IL-10 sequential pathway.. J Leukoc Biol.

[10] Tajima T, Murata T, Aritake K, Urade Y, Hirai H (2008). Lipopolysaccharide induces macrophage migration via prostaglandin D(2) and prostaglandin E(2).. J Pharmacol Exp Ther.

[11] Monteiro MC, Lima HC, Souza AA, Titus RG, Romao PR (2007). Effect of Lutzomyia longipalpis salivary gland extracts on leukocyte migration induced by Leishmania major.. Am J Trop Med Hyg.

[12] Teixeira CR, Teixeira MJ, Gomes RB, Santos CS, Andrade BB (2005). Saliva from Lutzomyia longipalpis induces CC chemokine ligand 2/monocyte chemoattractant protein-1 expression and macrophage recruitment.. J Immunol.

[13] Bozza PT, Magalhaes KG, Weller PF (2009). Leukocyte lipid bodies - Biogenesis and functions in inflammation.. Biochim Biophys Acta.

[14] Prates DB, Santos LD, Miranda JC, Souza AP, Palma MS (2008). Changes in amounts of total salivary gland proteins of Lutzomyia longipallpis (Diptera: Psychodidae) according to age and diet.. J Med Entomol.

[15] Plotkowski MC, Brandao BA, de Assis MC, Feliciano LF, Raymond B (2008). Lipid body mobilization in the ExoU-induced release of inflammatory mediators by airway epithelial cells.. Microb Pathog.

[16] Pacheco P, Bozza FA, Gomes RN, Bozza M, Weller PF (2002). Lipopolysaccharide-induced leukocyte lipid body formation in vivo: innate immunity elicited intracellular Loci involved in eicosanoid metabolism.. J Immunol.

[17] Lowry OH, Rosebrough NJ, Farr AL, Randall RJ (1951). Protein measurement with the Folin phenol reagent.. J Biol Chem.

[18] Laemmli UK (1970). Cleavage of structural proteins during the assembly of the head of bacteriophage T4.. Nature.

[19] Bozza PT, Melo RC, Bandeira-Melo C (2007). Leukocyte lipid bodies regulation and function: Contribution to allergy and host defense.. Pharmacol Ther.

[20] Brock TG, Peters-Golden M (2007). Activation and regulation of cellular eicosanoid biosynthesis.. ScientificWorldJournal.

[21] Giroux M, Descoteaux A (2000). Cyclooxygenase-2 expression in macrophages: modulation by protein kinase C-alpha.. J Immunol.

[22] Peters NC, Egen JG, Secundino N, Debrabant A, Kimblin N (2008). In vivo imaging reveals an essential role for neutrophils in leishmaniasis transmitted by sand flies.. Science.

[23] Oliveira SH, Canetti C, Ribeiro RA, Cunha FQ (2008). Neutrophil migration induced by IL-1beta depends upon LTB4 released by macrophages and upon TNF-alpha and IL-1beta released by mast cells.. Inflammation.

[24] Sa-Nunes A, Bafica A, Lucas DA, Conrads TP, Veenstra TD (2007). Prostaglandin E2 is a major inhibitor of dendritic cell maturation and function in Ixodes scapularis saliva.. J Immunol.

[25] Monteiro MC, Nogueira LG, Almeida Souza AA, Ribeiro JM, Silva JS (2005). Effect of salivary gland extract of Leishmania vector, Lutzomyia longipalpis, on leukocyte migration in OVA-induced immune peritonitis.. Eur J Immunol.

[26] Yu W, Bozza PT, Tzizik DM, Gray JP, Cassara J (1998). Co-compartmentalization of MAP kinases and cytosolic phospholipase A2 at cytoplasmic arachidonate-rich lipid bodies.. Am J Pathol.

[27] Moreira LS, Piva B, Gentile LB, Mesquita-Santos FP, D'Avila H (2009). Cytosolic phospholipase A2-driven PGE2 synthesis within unsaturated fatty acids-induced lipid bodies of epithelial cells.. Biochim Biophys Acta.

[28] D'Avila H, Melo RC, Parreira GG, Werneck-Barroso E, Castro-Faria-Neto HC (2006). Mycobacterium bovis bacillus Calmette-Guerin induces TLR2-mediated formation of lipid bodies: intracellular domains for eicosanoid synthesis in vivo.. J Immunol.

[29] Accioly MT, Pacheco P, Maya-Monteiro CM, Carrossini N, Robbs BK (2008). Lipid bodies are reservoirs of cyclooxygenase-2 and sites of prostaglandin-E2 synthesis in colon cancer cells.. Cancer Res.

[30] West MA, Clair L, Bellingham J, Wahlstrom K, Rodriguez JL (2000). Defective lipopolysaccharide-dependent ERK 1/2 activation in endotoxin tolerant murine macrophages is reversed by direct protein kinase C stimulation.. Shock.

[31] D'Avila H, Maya-Monteiro CM, Bozza PT (2008). Lipid bodies in innate immune response to bacterial and parasite infections.. Int Immunopharmacol.

[32] Bozza PT, D'Avila H, Almeida PE, Magalhães KG, Molinaro R (2009). Lipid droplets in host–pathogen interactions.. Clinical Lipidology.

[33] Afonso L, Borges VM, Cruz H, Ribeiro-Gomes FL, DosReis GA (2008). Interactions with apoptotic but not with necrotic neutrophils increase parasite burden in human macrophages infected with Leishmania amazonensis.. J Leukoc Biol.

[34] Matte C, Maion G, Mourad W, Olivier M (2001). Leishmania donovani-induced macrophages cyclooxygenase-2 and prostaglandin E2 synthesis.. Parasite Immunol.

[35] Anstead GM, Chandrasekar B, Zhao W, Yang J, Perez LE (2001). Malnutrition alters the innate immune response and increases early visceralization following Leishmania donovani infection.. Infect Immun.

[36] Barral A, Honda E, Caldas A, Costa J, Vinhas V (2000). Human immune response to sand fly salivary gland antigens: a useful epidemiological marker?. Am J Trop Med Hyg.

[37] Gomes RB, Brodskyn C, de Oliveira CI, Costa J, Miranda JC (2002). Seroconversion against Lutzomyia longipalpis saliva concurrent with the development of anti-Leishmania chagasi delayed-type hypersensitivity.. J Infect Dis.

[38] Souza AP, Andrade BB, Aquino D, Entringer P, Miranda JC Using recombinant proteins from Lutzomyia longipalpis saliva to estimate human vector exposure in visceral Leishmaniasis endemic areas.. PLoS Negl Trop Dis.

[39] Teixeira C, Gomes R, Collin N, Reynoso D, Jochim R Discovery of markers of exposure specific to bites of Lutzomyia longipalpis, the vector of Leishmania infantum chagasi in Latin America.. PLoS Negl Trop Dis.

